# Exploring the Burden of Knee Osteoarthritis in Rural South India: Community Prevalence, Risk Factors, and Functional Assessment Among Adults Aged 40 and Above

**DOI:** 10.7759/cureus.73452

**Published:** 2024-11-11

**Authors:** Saravanan Kandasamy, Rajalakshmi Vadivelu, Sathish Kumar Shanmugam, Balaji S Mahendran

**Affiliations:** 1 Department of Community Medicine, Government Pudukkotai Medical College, Pudukottai, IND; 2 Department of Physiology, Government Pudukkotai Medical College, Pudukottai, IND; 3 Department of Physiology, Government Medical College, Tiruppur, Tiruppur, IND; 4 Department of Community Medicine, Saveetha Medical College and Hospital, Saveetha Institute of Medical and Technical Sciences, Chennai, IND

**Keywords:** community prevalence, functional assessment, knee osteoarthritis, knee pain, womac

## Abstract

Background

The rising prevalence of osteoarthritis (OA) will create significant challenges for low-resource public health systems in countries like India, leading to increased disability and reduced quality of life. Limited access to imaging and specialized orthopedic care in rural areas often delays diagnosis and treatment until the disease has progressed to advanced stages, further burdening the economy.

Objectives

To estimate the community prevalence and assess the risk factors associated with OA among adults aged 40 and older in rural areas of South India and to determine their functional assessment among OA-diagnosed patients.

Methods

A community-based cross-sectional study was conducted in rural Tiruvallur district, Tamil Nadu, South India, from September 2017 to August 2018 among 427 people aged 40 and above. People with rheumatoid arthritis, reactive arthritis, old trauma of knee joint, and who have undergone total knee replacement were excluded from the study. A validated semi-structured questionnaire was used which consisted of the socio-demographic profile of the individuals, risk factors of knee OA, American College of Rheumatology (ACR) criteria for diagnosis of knee OA, Western Ontario and McMaster Universities Arthritis Index (WOMAC) scale and Visual Analog Scale (VAS) for functional assessment. A chi-square test and odds ratio were used to determine the significant risk factors and multivariate binomial logistic regression was performed to identify the predictors of knee OA.

Results

The mean age of the study population was 60 years ranging from 42 to 89 years with 48% males and 52% females. The community prevalence of knee OA was 34.6%. The significant predictors for the development of OA were diabetes, obesity, Indian toilet use, and hypertension in women. The level of pain among OA patients was higher for standing from a sitting position and while climbing upstairs. The mean WOMAC score of OA subjects was 57.38 (± 12.16) ranging from 25 to 78.

Conclusion

The study reveals a high prevalence of knee OA among over one-third of individuals aged 40 and above in rural South India, with significant risk factors including obesity, diabetes, hypertension, menopause, sedentary lifestyles, and Indian-style toilets. Severe functional impairment, indicated by high WOMAC scores, underscores limited healthcare access, emphasizing the need for community interventions in lifestyle modification and chronic disease management.

## Introduction

Osteoarthritis (OA) is a chronic degenerative joint disease characterized by cartilage deterioration and osteophyte (abnormal bony growth) formation, particularly affecting weight-bearing joints like the knees. OA knee, a common form of arthritis, is primarily attributed to wear and tear on the joints. This chronic condition, with multifactorial origins, results in structural and biochemical changes in the synovial membrane and capsule of the affected joints [1]. While primary OA is often age-related and idiopathic, secondary OA can be triggered by underlying diseases or conditions. The pathological progression of OA involves softening, ulceration, and disintegration of the articular cartilage, accompanied by inflammation of the synovial membrane [2]. Typical clinical symptoms are pain, sometimes swelling particularly after prolonged activity and weight-bearing; whereas stiffness after inactivity. It is also known as degenerative arthritis, it usually affects the large weight-bearing joints, such as the hips, knees hands, feet, and spine [2].

OA is the second most common rheumatologic issue globally, with a reported prevalence of 14% to 18% among adults and 20% to 27% among 40 years and older [3]. Various studies in India reported the prevalence of OA around 20% to 40% [4-8]. A community-based cross-sectional study by Pal et al. across pan India estimated the prevalence of knee OA as 28.7% where OA was diagnosed using the Kellgren and Lawrence scale [4]. Jadhao et al. used ACR criteria for diagnosing OA and deduced a prevalence of 34.7% in rural Maharashtra, India [7]. Venkatachalam et al. also diagnosed OA by ACR criteria and estimated prevalence of 27.1% in rural Tamil Nadu, South India [8]. Women are more commonly affected by OA, with prevalence increasing with age. The burden of OA on mobility is significant, particularly among females, and the disease becomes more prevalent in women after the age of 45 [9,10].

Various risk factors contribute to the development of significant knee OA, including advancing age, female gender, overweight/obesity, occupational factors like knee-bending postures and physical labor, genetic predisposition, joint trauma, vitamin D deficiency, and metabolic disorders like chondrocalcinosis [5]. Lifestyle factors such as lack of exercise, obesity, and conditions like diabetes exacerbate the disease. Inflammatory diseases, improper postures, and trauma further complicate the condition, especially in females during the peri-menopausal period [11].

The rapid increase in the prevalence of OA will lead to a situation where the disability and poor quality of life associated will pose a major challenge to evolving low-resource public health systems like in India and place a major burden on the economy of the country. Functional assessment is crucial in evaluating the impact of OA on daily living and is vital for developing effective treatment strategies to improve a patient's quality of life. The lack of imaging facilities and specialized orthopedic care in rural India causes a deferral in diagnosis and treatment until the late stages of the disease process [6].

Despite the known risk factors and prevalence of OA, there is limited evidence on the impact of co-morbidities, lifestyle factors, and socio-demographic variables on OA burden. Investigating the current burden of OA and its association with various factors in different socio-cultural settings is essential for developing tailored management approaches. Therefore, this study aims to explore the prevalence of knee OA and their risk factors in the Indian population especially among adults aged 40 and above, focusing on rural settings to address this research gap and the functional assessment of OA patients for understanding their impact on daily living.

## Materials and methods

Study setting and population

A community-based cross-sectional study was conducted in Vilangadupakkam, a rural area of Tiruvallur district, Tamil Nadu, South India from September 2017 to August 2018. Vilangadupakkam area, a rural field practice area of Stanley Medical College, comprises a population of 3,598 adults of 40 years and above with 1,819 males and 1,779 females. The study population consisted of households living in selected houses in the study area. People aged 40 and above of both genders were included in the study. People with rheumatoid arthritis, reactive arthritis, old trauma of knee joint, and who have undergone total knee replacement were excluded from the study.

Sample size

According to Pal et al.'s study, considering the prevalence of OA among adults over 40 years (p) as 28.7%, with a precision (d) of 4.5%, at 95% confidence interval (Z₁₋ₐₗ₂ = 1.96), the sample size is calculated using the formula, N = Z²₁₋ₐₗ₂ * p * (1 - p) / d² = 1.96² * 0.287*(1 - 0.287)/0.045² = 388 [4]. Considering a 10% non-response rate, N = 388 + 0.1(388) = 427. Thus, the total sample size required for the study was 427.

Study tool

A validated semi-structured questionnaire was used for the data collection. The questionnaire consisted of three parts: I part - socio-demographic profile of the individuals and risk factors (medical history and lifestyle factors) of knee OA, II part - Diagnosis of knee OA by clinical examination using American College of Rheumatology (ACR) criteria and III part - Functional assessment of knee OA using Western Ontario and McMaster Universities Arthritis Index (WOMAC) scale and Visual Analog Scale (VAS) for measuring level of pain and history of type of pain they experience.

The clinical ACR criteria for knee OA are chronic pain in the knee and at least three of the following: age > 50 years, stiffness < 30 min, crepitus, bony tenderness, bony enlargement, and no palpable warmth [12]. The first author was a trained orthopedic surgeon who clinically examined all the subjects for the diagnosis of knee OA. WOMAC consists of 24 items of a self-administered questionnaire divided into three subscales of pain (five items), stiffness (two items), and physical function (17 items) scored on a Likert scale of 0-4 with a maximum score of 96 which is transformed to percentage. Higher scores increase the level of symptoms and physical disability [13]. The WOMAC questionnaire was freely available and used for the study.

Sampling and data collection

The study subjects were selected by systematic random sampling. The study sample size was 427 so the sampling interval was determined as 3,598/427=8. The first house to be started was randomly selected as third house by lot method. Thereafter, every eighth house was selected at regular intervals. One eligible participant was selected randomly by the last birthday method and interviewed from each house after written informed consent. The next house was selected if the selected house was locked on two consecutive visits. The data from the participants was collected using the questionnaire along with a clinical examination of the patient after obtaining written informed consent. Prior permission was obtained from the Village head and local village health nurses aided the community involvement in the process of data collection. The Institutional Ethics Committee approval was obtained from the Stanley Medical College before the conduct of the study (IEC No: 21205/2016).

Statistical analysis

The data collected were analyzed using IBM SPSS (Statistical Product and Service Solutions) version 21 (IBM Corp., Armonk, NY). The categorical data were expressed as frequency and percentages while continuous data as mean and standard deviation. A chi-square test was performed to determine the significant risk factors associated with knee OA and the odds ratio was used to measure the strength of the association. Multivariate Binomial logistic regression analysis was performed to identify the predictors of knee OA and measured their strength of association with the adjusted odds ratio.

## Results

The mean age of the study population was 60 years ranging from 42 to 89 years with 48% males and 52% females. The study participants consisted of 82.6% Hindus, 13.8% Christian, and 3.5% Muslims. More than one-third of the population (35%) had no formal education, 28% had completed primary school, 15.6% in middle school, and 10.5% in high school. 24% of the participants were unemployed and 60.5% were doing unskilled jobs. Almost 80% belonged to the lower (11%) and upper lower class (69%).

The comorbidity pattern of the study subjects was as follows; 6.5% were obese, 14.3% were diabetic and 13% were hypertensive. 8.9% had tobacco consumption and 0.7% had alcohol consumption. Among the tobacco consumption, the majority were smokers (68%) and others had tobacco chewing. 33.3% of the women in the study population attained menopause. 28% had sedentary physical activity and 65% of them had sitting type of work. 63.2% were using the Indian type of toilet for defecation (Table 1).

**Table 1:** Demographic, comorbidities, and lifestyle factors of the study population # - expressed in Mean (± standard deviation) and range BMI ≥ 30 kg/m^2^ was classified as obese, BMI ≥ 25 and < 30 kg/m^2^ as overweight and BMI of 18.5 - 24.9 kg/m^2^ as normal as per WHO guidelines

		Frequency (n = 427)	Percentage
Age (years)^#^		60.05 (± 11)	42-89
Gender	Males	205	48.01
	Females	222	51.99
Religion	Hindu	353	82.67
	Christian	59	13.82
	Muslim	15	3.51
Education	No formal education	150	35.13
	Primary school	120	28.10
	Middle school	67	15.69
	High school	45	10.54
	Intermediate/diploma	24	5.62
	Graduate	21	4.92
Occupation	Unemployed	103	24.12
	Unskilled	258	60.42
	Semi-skilled	34	7.96
	Skilled	13	3.04
	Clerical job	19	4.45
Socio-economic status	Lower class	48	11.24
(Modified Kuppusamy Scale)	Upper lower class	293	68.62
	Lower middle class	71	16.63
	Upper middle class	15	3.51
BMI	Obese	28	6.56
	Overweight	181	42.39
	Normal	218	51.05
Diabetes mellitus		61	14.29
Duration of diabetes (years)^#^		3.62 (± 3.19)	1-15
Hypertension		56	13.11
Duration of hypertension (years)^#^		3.68 (± 3.26)	1-20
Tobacco consumption		38	8.90
Tobacco use pattern	Smoking	26	68.42
	Chewing	12	31.58
Alcohol consumption		3	0.70
Attained menopause (women)		74/222	33.3
Type of physical activity	Sedentary	119	27.87
	Moderate	308	72.13
Common work position	Prolonged standing	150	35.12
	Sitting	277	64.87
Type of toilet use	Indian type	270	63.23
	Western type	157	36.77

Among the study population, knee OA was seen in 148 (34.6%) subjects. This prevalence of 34.6% indicates the community prevalence of knee OA among 40-year-olds and above in rural areas.

The demographic and lifestyle factors were analyzed for their association with OA of the knee. The analysis revealed that obesity (OR: 5.29, CI: 2.27-12.3), diabetes mellitus (OR: 16.25, CI: 7.71-34.2), hypertension (OR: 5.01, CI: 2.74-9.16), tobacco consumption (OR: 2.27, CI: 1.16-4.43), alcohol consumption (OR: 11.54, CI: 0.6-232.0), attained menopause in women (OR: 21.54, CI: 12.2-38.0), a sedentary lifestyle (OR: 3.26, CI: 2.1-5.07), prolonged sitting (OR: 2.51, CI: 1.6-3.9), and the use of Indian type toilets (OR: 12.26, CI: 6.5-23.1) were significantly associated with a higher risk of OA. Other factors, such as age, gender, education level, employment status, and socio-economic status, did not show significant associations with OA (Table 2).

**Table 2:** Comparison of demographic, comorbidities, and lifestyle factors with knee OA P-value determined by Chi-square test. Fisher Exact test was performed when the expected value was less than 5. C.I. - 95% confidence interval for odds ratio. OA: osteoarthritis

		Osteoarthritis knee				P-value	Odds ratio (C.I.)
		Yes (n = 148)		No (n = 279)			
		n	%	n	%		
Age	60 years & above	69	35.6	125	64.4	0.719	1.08 (0.72-1.61)
	Less than 60 years	79	33.9	154	66.1		
Gender	Males	64	31.2	141	68.8	0.151	0.75 (0.5-1.11)
	Females	84	37.8	138	62.2		
Education	Educated	88	31.8	189	68.2	0.088	0.7 (0.46-1.06)
	No formal education	60	40.0	90	60.0		
Occupation	Employed	108	33.3	216	66.7	0.307	0.79 (0.5-1.25)
	Unemployed	40	38.8	63	61.2		
Socio-economic status	Lower classes	125	36.7	216	63.3	0.084	1.59 (0.94-2.68)
	Middle classes	23	26.7	63	73.3		
Obesity	Yes	20	71.4	8	28.6	0.001	5.29 (2.27-12.3)
	No	128	32.1	271	67.9		
Diabetes Mellitus	Yes	52	85.2	9	14.8	0.001	16.25 (7.71-34.2)
	No	96	26.2	270	73.8		
Hypertension	Yes	38	67.9	18	32.1	0.001	5.01 (2.74-9.16)
	No	110	29.6	261	70.4		
Tobacco consumption	Yes	20	52.6	18	47.4	0.015	2.27 (1.16-4.43)
	No	128	32.9	261	67.1		
Tobacco use pattern	Smoking	8	30.8	18	69.2	0.001	0.02 (0-0.35)
	Chewing	12	100.0	0	0.0		
Alcohol consumption	Yes	3	100.0	0	0.0	0.041	11.54 (0.6-232.0)
	No	145	34.2	279	65.8		
Attained Menopause (Women)	Yes	61	82.4	13	17.6	0.001	25.5 (12.1-53.7)
	No	23	15.5	125	84.5		
Type of physical activity	Sedentary	65	54.6	54	45.4	0.001	3.26 (2.1-5.07)
	Moderate	83	26.9	225	73.1		
Common work position	Sitting	115	41.5	162	58.5	0.001	2.52 (1.6-3.9)
	Prolonged standing	33	22.0	117	78.0		
Type of toilet use	Indian type	136	50.4	134	49.6	0.001	12.26 (6.5-23.1)
	Western type	12	7.6	145	92.4		

The significant predictors for the development of OA were Indian toilet use (AOR: 10.56, CI: 5.3-21.2), obesity (AOR: 8.03, CI: 2.81-22.94), diabetes (AOR: 13.44, CI: 5.77-31.31), and hypertension (AOR: 2.69, CI: 1.28-5.67). Even though sedentary physical activity and sitting position were significant they had collinearity with obesity and hence not included in the model. Hosmer Lemeshow test for goodness of fit had a p-value of 0.779 indicating the model was fit with a maximum likelihood of 0.462 (Nagelkerke R square) (Table 3).

**Table 3:** Multivariate binomial logistic regression for predicting knee OA C.I. - 95% confidence interval; AOR - Adjusted Odds Ratio; B - regression coefficient (Beta); S.E. - standard error of Beta; df - degree of freedom; OA - osteoarthritis

Predictors for osteoarthritis	B	S.E.	Wald	df	P-value	Adjusted odds ratio	95% C.I. for AOR	
							Lower	Upper
Indian toilet use	2.357	0.356	43.88	1	0.001	10.56	5.25	21.21
Obese	2.083	0.536	15.10	1	0.001	8.03	2.81	22.94
Diabetes	2.598	0.431	36.27	1	0.001	13.44	5.77	31.31
Hypertension	0.991	0.380	6.82	1	0.009	2.69	1.28	5.67
Constant	-3.033	0.341	79.18	1	0.001	0.05		

Among the OA subjects, symptomatically, swelling pain was the most common, seen in 56.1% of the patients, followed by grinding pain (54.7%), catch pain (52%), and clicking pain (41.9%). The mean level of pain was during standing from sitting position and while going upstairs which was above 6.5 ranging from 3 to 8. The mean level of pain during other activities varied from 6 to 6.5 ranging from 0 to 8. The mean WOMAC score of OA subjects was 57.38% (± 12.16%) ranging from 25% to 78% (Table 4).

**Table 4:** Functional assessment of knee OA patients S.D. - standard deviation; OA - osteoarthritis

Functional assessment (n = 148)			
Type of pain felt in knee	Often	Rarely	Never
Swelling pain	83 (56.1%)	8 (5.4%)	57 (38.5%)
Clicking pain	62 (41.9%)	20 (13.5%)	66 (44.6%)
Catch pain	77 (52%)	21 (14.2%)	50 (33.8%)
Grinding pain	81 (54.7%)	17 (11.5%)	50 (33.8%)
VAS score – level of pain	Mean	S.D.	Range
Standing from sitting position	6.55	1.51	3-8
While going upstairs	6.51	1.50	3-8
While going downstairs	6.34	1.64	0-8
While straightening	6.32	1.75	0-8
While bending knee	6.34	1.76	0-8
While getting on/off toilet	6.26	1.77	0-8
WOMAC score – level of disability	Mean	S.D.	Range
WOMAC score	57.38	12.16	25-78

## Discussion

The study identified a knee OA prevalence of 34.6% among individuals aged 40 and above in the rural community. This significant prevalence highlights the considerable burden of knee OA in rural areas of South India, particularly among older adults. The finding is consistent with other research conducted in similar settings. Jadhao et al. (34.7%) and Bala et al. (35.7%) found knee OA prevalence of 34.7% among more than 40 years in rural areas in North India [7,14]. Pal et al. showed a prevalence of 28.7% in a multicentric study in India [4].

The present study shows that obesity was significantly associated with knee OA with an odds ratio (OR) of 5.29 (CI: 2.27-12.3). Multiple studies have found significant odds ratios for this relationship, ranging from 2.45 to 5.24 for obese individuals compared to those of normal weight [15,16]. A meta-analysis by Zheng et al. revealed that the risk of knee OA increases by 35% for every 5 kg/m² increase in BMI [15].

Diabetes had a strong association with knee OA with an odds of 16.25. A systematic review and meta-analysis by Williams et al. found that T2DM was associated with the development and presence of OA, even when controlling for body mass index [17]. In Nieves-Plaza et al.'s study on a Hispanic population from Puerto Rico, Diabetics had 2.18 times higher odds of having hand or knee OA after adjusting for age and sex [18]. Jadhao et al.'s study also found a significant association of diabetes with knee OA among the elderly [7]. Duclos's study suggests that both obesity and DM increase OA risk through mechanical and metabolic pathways, including adipokine production and insulin resistance [19].

The current study implies hypertension as a significant risk factor for knee OA with an odds of 5. A longitudinal study by Veronese et al. found that individuals with knee OA had a 13% higher risk of developing hypertension [20]. A meta-analysis by Lo et al. has reported that hypertension is associated with increased odds of both radiographic and symptomatic knee OA and is independent of BMI. These findings suggest a potential vascular etiology for OA [21].

The current study deciphered that 82.4% of menopause-attained women had knee OA with an odds of 21.4, which was supported by evidence from many studies [22-24]. Postmenopausal women exhibit more severe knee joint cartilage degeneration compared to pre- and perimenopausal women, due to estrogen deficiency, with progressive degeneration occurring in the first 25 years after menopause [24]. Early menopause, occurring before age 40, is linked to early-onset KOA, with symptoms developing within one to two years post-menopause [22,23].

Almost half of the population with Sedentary physical activity and prolonged sitting had significant odds (3.26 and 2.51, respectively) of developing knee OA. Sedentary behavior was prevalent among 69.2% of knee OA patients in a cross-sectional study by Ez-zaoui et al., with factors such as age, pain, and functional disability associated with it [25]. A large cohort study by Voinier et al. found that leisure time sitting for >4 hours/day was linked to a 25% higher risk of radiographic knee OA incidence/progression over two years, with the risk further intensified by frequent worktime sitting [26]. This sedentary behavior and prolonged sitting contribute to obesity which further aggravates the development of knee OA.

The use of Indian type of toilets for defecation was significantly associated with knee OA with an odds of 12.26. Prolonged squatting was identified as a strong risk factor for tibiofemoral knee OA in elderly Chinese subjects by Zhang et al. [27]. Similarly, habitual squatting, lotus position, and side-knee bending were found to increase the risk of knee OA in the Thai population [28]. The use of regular toilets, as opposed to Western-style toilets, was significantly associated with knee OA in an Iranian study [29]. These findings suggest that floor activities and toilet types common in Asian countries may contribute to the development of knee OA.

Certain studies mention increasing age and female gender were more associated with knee OA [4,30], but the current study did not elucidate any significant association between age and gender and also for other demographic characteristics like education, occupation, and socio-economic status. Considering all the factors together, Diabetes, Obesity, hypertension, and Indian toilet use were the significant predictors of knee OA with significantly adjusted odds ratio as deciphered from the Binomial logistic regression.

Symptomatically, swelling pain was the most common, affecting 56.1% of patients, followed by catch pain (54.7%), clicking pain (52%), and grinding pain (41.9%). Swelling pain, often caused by inflammation in the joint, is commonly associated with synovial fluid buildup and cartilage degradation [31]. Catch pain, clicking, and grinding sensations are indicative of mechanical dysfunction within the joint, likely due to the breakdown of cartilage, resulting in bones rubbing together or catching during movement [32]. The high prevalence of these symptoms underscores the significant functional impairment that knee OA can cause, limiting mobility and affecting the quality of life.

In this study, the mean WOMAC score for OA subjects was 57.38% (± 12.16%), with scores ranging from 25% to 78%. This score reflects a moderate-to-severe level of symptom burden among the study population, indicating significant impairment in daily activities and overall quality of life. A study conducted by Creamer et al. on older adults with knee OA reported a mean WOMAC score of approximately 55%, suggesting a similar level of symptom burden [33]. However, some populations with more access to early treatment and preventive care may present with slightly lower WOMAC scores. The higher end of the range observed in this study, with scores reaching 78%, suggests that a significant proportion of patients experience severe disability and pain. The study population's rural setting may contribute to higher WOMAC scores due to limited access to healthcare services, physical therapy, and early intervention.

The current study is not without limitations. It was almost a cross-sectional study with limited area coverage which could reduce the generalizability of the findings. Since the determination of OA was done at the field level with self-reported data, there could be a minimal level of measurement bias compared to done in a hospital setting. The strength of the study is it clearly mentioned the risk factors for knee OA and its predictability with odds of the predictors. Also, it highlighted the estimate of absenteeism due to knee OA.

Given the high prevalence of knee OA, awareness programs are essential to educate rural populations on knee OA risk factors, symptoms, and preventive measures. Early recognition of symptoms and understanding of lifestyle adjustments could help in managing and reducing the disease burden. Since obesity and diabetes significantly increase the risk of knee OA, integrating weight management programs and diabetes control initiatives into public health services in rural areas can help mitigate these risk factors. Regular screening for BMI and blood glucose levels, alongside tailored interventions, may reduce the incidence and severity of knee OA. Community-based physical activity programs or structured exercise regimens promoting active lifestyles, especially targeting those with sedentary work or leisure habits, to reduce prolonged sitting and improve joint health could reduce knee OA risk.

Introducing affordable, accessible alternatives to squatting toilets and providing subsidies or community grants for installing Western-style toilets may be beneficial and could reduce strain on knee joints, particularly among older adults. Postmenopausal women represent a high-risk group for knee OA. Healthcare providers should focus on hormone replacement therapy (HRT) education, lifestyle counseling, and knee-strengthening exercises, specifically tailored to postmenopausal women, to slow OA progression. To address the functional impairments and high symptom burden, improving access to pain management, physical therapy, and early intervention services in rural areas is crucial.

## Conclusions

The study highlights the substantial prevalence and burden of knee OA among more than one-third of individuals aged 40 and above in rural South India. Key predictors, including obesity, diabetes, hypertension, sedentary lifestyle, attained menopause, and Indian-style toilets, were significantly associated with increased knee OA risk. The high WOMAC scores observed reflect severe impairment and highlight the limited healthcare access in these communities, which may exacerbate disease impact and quality of life. The findings emphasize the need for targeted community interventions focusing on lifestyle modification, diabetes, and obesity management, and improved healthcare access to mitigate the impact of knee OA. Tailoring these recommendations to local needs and available resources could significantly alleviate the burden of knee OA and improve the overall health and quality of life in these rural populations.

## Appendices

Questionnaire

1.Subject ID. No.: _______

2.Name: ____________________________________

3.Age: ___________ years

4.Gender: 1. Male 2. Female 3. Transgender

5.Address: _______________________________________________________

6.Mobile Number: ___________________________

7.Area: 1. Urban 2. Rural

8.Religion: 1. Hindu 2. Chirstian 3. Muslim 4. Others

9.Education: 1. Profession or Honours 2. Graduate or Post graduate 3. Intermediate or Post high school diploma 4. High school certificate 5.Middle School certificate 6. Primary school Certificate 7. Illiterate

10. Occupation: 1. Professional 2. Semi-professional 3. Clerical, shop-owner, farmer 4. Skilled worker 5. Semiskilled worker 6. Unskilled worker 7. Unemployed

11. Family Income (per month): _______ Rs.

12. Socioeconomic status (B.G Prasad scale per-capita family income): 1. Upper 2. Upper middle 3. Middle 4.  lower Middle 5. Lower

13. Height (in cm): ________

14. weight (in kg): ________

15.BMI (body mass index): 1. Underweight 2. Normal 3. Overweight 4. Obese

16. Are you a Diabetic: 1. Yes 2. No

17. How long are you a Diabetic: _____ years

18. Are you a Hypertensive: 1. Yes 2. No

19. How long are you a hypertensive: _____ years

20. Do you use tobacco? 1.yes 2. No

21. Tobacco use pattern: 1. Smoking 2. Chewing 3. Sniffing 4. Others

22. Do you consume alcohol: 1. Yes 2. No

23. Attained Menopause (Women): 1. Yes 2. No

24. Type of physical activity: 1. Sedentary 2. Moderate 3. Heavy

25. Common work positions: 1. Prolonged standing 2. Sitting

26. Type of toilet use: 1. Indian 2. Western 3. Both Knee OA patients

27. Clinical examination of knee joint: Right knee joint / Left knee joint

      1 Swelling

      2 Deformity

      3 Tenderness

      4 Crepitus

      5 Range of movements

28. Type of Pain in Knee 1. Swelling pain 2. Clicking pain 3. Catching pain 4. Grinding pain

29. Level of Pain in Knee - VAS score 1 2 3 4 5 6 7 8 9 10

      1 Level of pain standing from sitting

      2 Level of pain while going up stairs

      3 Level of pain while going downstairs

      4 Level of pain while straightening knee

      5 Level of pain while bending knee

      6 Level of pain while getting on/off toilet

30. WOMAC scale
